# Cell-free DNA technologies for the analysis of brain cancer

**DOI:** 10.1038/s41416-021-01594-5

**Published:** 2021-11-22

**Authors:** Richard Mair, Florent Mouliere

**Affiliations:** 1 Cancer Research UK Cambridge Institute, University of Cambridge, CB2 0RE Cambridge, UK; 2 Cancer Research UK Major Centre – Cambridge, Cancer Research UK Cambridge Institute, CB2 0RE Cambridge, UK; 3 Division of Neurosurgery, Department of Clinical Neurosciences, University of Cambridge, CB2 0QQ Cambridge, UK; 4 Amsterdam UMC, Vrije Universiteit Amsterdam, Department of Pathology, Cancer Centre Amsterdam, 1081 HV Amsterdam, The Netherlands

**Keywords:** CNS cancer, Next-generation sequencing, Biomarkers

## Abstract

Survival for glioma patients has shown minimal improvement over the past 20 years. The ability to detect and monitor gliomas relies primarily upon imaging technologies that lack sensitivity and specificity, especially during the post-surgical treatment phase. Treatment-response monitoring with an effective liquid-biopsy paradigm may also provide the most facile clinical scenario for liquid-biopsy integration into brain-tumour care. Conceptually, liquid biopsy is advantageous when compared with both tissue sampling (less invasive) and imaging (more sensitive and specific), but is hampered by technical and biological problems. These problems predominantly relate to low concentrations of tumour-derived DNA in the bloodstream of glioma patients. In this review, we highlight methods by which the neuro-oncological scientific and clinical communities have attempted to circumvent this limitation. The use of novel biological, technological and computational approaches will be explored. The utility of alternate bio-fluids, tumour-guided sequencing, epigenomic and fragmentomic methods may eventually be leveraged to provide the biological and technological means to unlock a wide range of clinical applications for liquid biopsy in glioma.

## Introduction

Gliomas are rare and deadly brain pathologies that possess an age-corrected incidence range of 4.67–5.73 per 100,000 persons [1] with glioblastoma being the most common malignant primary brain tumour [2]. The current treatment paradigm involves maximal surgical resection (where possible) followed by radiotherapy and chemotherapy (temozolomide). Complete resection of contrast-enhancing tumour as demonstrated on magnetic resonance imaging (MRI) improves average survival, which, with the addition of chemotherapy and radiotherapy, is just over 1 year [3]. The location of this cancer within the brain results in certain patients being unsuitable for ‘complete’ resection. For these cases, biopsy is performed to confirm the diagnosis if further treatment (chemotherapy and radiotherapy) is indicated, but this carries significant risk of neurological morbidity and mortality. Despite treatment, only 30% of patients survive their first year after diagnosis and only 13% survive for 5 years as patients often present late and with extensive disease.

Liquid biopsy may have utility across all phases of brain-tumour investigation and management. Although screening for rare pathologies is difficult, a non-invasive, low-cost and reliable cancer- diagnostic assay could potentially benefit cancer patients and the public healthcare system. If glioma were to be included, then this may provide some benefit for certain glioma patients. Diagnosis of glioma, especially in patients for whom debulking surgery is not indicated, would benefit from minimally invasive diagnostic options such as liquid biopsy. Moreover, the ability to characterise the heterogeneous genomic landscape may be of further benefit for precision therapeutics. The expectation for disease recurrence following first-line treatment for glioma mandates the use of regular clinical follow-up associated with interval contrast-enhanced MRI. This paradigm has limitations with several radiological scenarios, such as pseudoprogression, an abnormal contrast enhancement associated with improved prognosis that affects up to one-third of patients, highlighting the need for improved detection and monitoring protocols. The frequency with which patients are currently monitored is dictated by pragmatism related to logistics and cost [4]. MR imaging mandates specialist equipment, trained individuals, comes at high cost per patient and possesses uncertainties with regard to the frequent use of gadolinium contrast. Existing radiological and clinical strategies lack sensitivity and specificity, leading to uncertainty as to when to stop therapies that lack efficacy, and when to persevere. Monitoring of tumour burden and treatment response with an effective liquid-biopsy approach may help to address these challenges [5, 6].

Multiple analytes can be detected in the bio-fluids of patients with cancer and used as liquid biopsy for a range of clinical applications: cell-free DNA (cfDNA), cell-free RNA, mitochondrial DNA, extracellular vesicles, tumour-educated platelets, proteins and metabolites among others [7–9]. cfDNA analysis, in particular, has emerged over the past years as potential game changer for detection, monitoring and treatment guidance in oncology [10, 11]. Despite the importance of investments from academic institutions and commercial entities, clinical validation has delayed widespread implementation [12]. Whilst initial data indicating that cfDNA may be challenging to detect using blood samples in glioma [13], several biological (e.g. impact of the blood–brain barrier) and technical reasons for this apparent difficultly exist. We aim to summarise recent progress in the application of liquid biopsy for brain-tumour characterisation and to identify future research directions.

## The concentration in plasma cfDNA can be increased for gliomas

Due to the technical limitations of the time, initial studies that recovered cfDNA focused on global DNA quantification in control and diverse malignant plasma samples [14]. The overall load of cfDNA molecules in plasma (or other bio-fluids) from patients with brain tumours can be recovered quickly and at minimal cost (via spectrofluorometer methods or PCR quantification) and has recently regained interest [15]. Multiple studies have explored the difference in concentration of cfDNA between various types of gliomas and healthy controls, alone or in combination with other methods [16–18]. In a prospective study, glioma patients had higher plasma cfDNA concentration than age-matched healthy controls prior to initial surgery (mean 13.4 vs. 6.7 ng/mL, respectively) and this correlated with tumour burden on pre-irradiation MRI [16]. Another study identified in patients with progressive disease, a significant increase in cfDNA concentration from pretreatment to time of progression (9.7 vs. 13.1 ng/mL, *p* = 0.037), while no difference was observed for non-progressive patients [18]. Despite global DNA-modification evaluation, using cfDNA concentration is likely to be hampered by a lack of specificity (as cfDNA can be released by various physiological mechanisms), which may limit clinical applicability [19].

## Detecting mutant cfDNA is challenging for gliomas

cfDNA is commonly detected in bio-fluids by analysing tumour-derived genomic signals, such as single- nucleotide variants (SNVs). SNVs due to their binary and potentially actionable nature, were logically the first molecular targets for cfDNA liquid-biopsy assays in the brain context. Early pan-cancer studies that evaluated cfDNA tumour fraction from various technologies have revealed that gliomas are the most challenging malignancy for liquid-biopsy applications [13, 20]. Using BEAming, a PCR-based technology, Bettegowda et al. detected mutant cfDNA in <10% of the plasma from 27 patients with gliomas [13]. Both the detection rate and concentration in mutant cfDNA were lower than those of the 14 other cancer types included in this study. Table 1 summarises the results reported from previous studies using SNVs to detect cfDNA in gliomas. Claims of higher rates of detection, and higher tumour fractions, have been made by entities using proprietary-sequencing assays [20–22]. The high frequency of alterations resulting from clonal haematopoiesis alongside biological or technical noise may, however, have confounded these results [23]. Locus-based assays (digital PCR, targeted sequencing or CRISPR-based) exhibit some potential to detect well-defined mutations at a low tumour fraction in the circulation [24–26]. In a recent study, TERT promoter mutations were evaluated in plasma using a custom digital- droplet PCR assay. An overall sensitivity of 62.5% (95% CI, 52–73%) and a specificity of 90% (95% CI, 80–96%) when compared with matched tumour-tissue-based detection was identified [27]. Nevertheless, these studies highlight that more sensitive methods, or alternative approaches, for plasma cfDNA detection, are required in gliomas.

**Table 1:** Summary of studies analysing SNVs and other genomic alterations in gliomas plasma samples.

Study	Technology	Method	Cohort	Results
Bettegowda et al. [13]	PCR-based	BEAming	Pan-cancer—among them 27 with GBM	10/27 detection rate
Schwaederle et al. [21]	Targeted sequencing	54-gene panel (Guarduant360)	Pan-cancer—among them 33 with GBM	24% with one alteration, 3% with two or more alterations
Zill et al. [20]	Targeted sequencing	54-gene panel (Guarduant360)	Pan-cancer—among them 107 with glioma/GBM	51% detection rate, median tumour fraction 0.45%
Piccioni et al. [22]	Targeted sequencing	54-gene panel (Guarduant360)	419 patients with primary brain cancer (various sub-types, 222 with GBM)	211/419 (50%) detection rate of at least 1 alteration. 55% detection rate for GBM
Bagley et al. [16]	Targeted sequencing	54-gene panel (Guarduant360)	20 patients with GBM	11/20 (55%) detection rate of at least 1 alteration. Mean tumour fraction 0.84%
Mouliere et al. [47]	Personalised sequencing	INVAR	8 patients with GBM	mutations were detected in 7/8 CSF, 10/12 plasma and 10/16 urine gliomas samples.
Pan et al. [37]	Capture sequencing	68-gene panel (Genetron Health)	8 patients with GBM	3/8 (37.5%) detection rate of at least 1 alteration in plasma.

### Sampling the cerebrospinal fluid as a liquid biopsy

The blood–brain barrier protects the central nervous system and has been proposed as the main reason for low cfDNA in glioma patients. Thus, a potential strategy for improving cfDNA yield may be through collecting samples ‘closer’ to the tumour. The most direct method by which to achieve this may be via cerebrospinal fluid (CSF) sampling [28–30]. Compared with blood plasma, CSF in glioma has shown higher relative tumour fraction although this is due, in part, to the low levels of normal DNA present within this immune-privileged anatomic location [31–33]. Studies examining brain-tumour patients (using whole-exome sequencing or PCR-based methods) have identified higher cfDNA tumour fraction in CSF when compared with plasma, and a higher detection rate [28, 29, 34]. The analysis of tumour-derived cfDNA in CSF from a cohort of diffuse gliomas indicated that they could be subtyped through mutational analysis with *IDH1* and *IDH2*, *ATRX*, *TP53*, *TERT*, and *H3F3A* being suggested as having classification and prognostication potential [35]. The representation of intra-tumoural heterogeneity is biased in CSF with shared clones being overrepresented, and private clones unrepresented, mimicking plasma observations in other cancer types [34]. Nevertheless, the high tumour fractions observed in CSF permit a higher detection range with multiple technologies ranging from PCR [29], to targeted sequencing [30, 35], low-coverage whole-genome sequencing (WGS) [36] and other genome-wide approaches [28, 33, 34]. For example, by using deep sequencing of cfDNA on CSF from 57 brain-tumour patients, at least one tumour-specific mutation was detected in over 82.5% of CSF ctDNA samples (47/57) [37]. In 8 selected cases with mutation detected in CSF, mutations were detected in 3/8 (37.5%) matched plasma samples [37]. This relatively high tumour fraction in CSF has allowed preliminary clinical application and molecular characterisation of gliomas [35, 37, 38]. Unfortunately use of CSF has significant clinical limitations as CSF is obtained via lumbar puncture, an invasive and morbid procedure relatively contraindicated in brain-tumour patients [39, 40]. CSF use in monitoring for recurrence is likely to be limited as a consequence. Moreover, despite the use of advanced sequencing and bioinformatic techniques, the tumour-derived cfDNA-detection rate in CSF remains relatively low, ranging from 39% to 98% (the highest detection rate being reported in brain metastasis from other primary sites) [33, 34]. Potential factors affecting this include the position of the tumour within the brain, whether the tumour is in direct contact with the CSF or not [29], and by the high level of intra-tumour heterogeneity in gliomas [41]. The acceptance and clinical integration of liquid biopsy for the management of gliomas will always be compared with the current imaging paradigm. Therefore, truly minimally invasive sampling, like blood plasma or urine, alongside better sensitivity and specificity, will be mandatory. Several emerging concepts and technologies have the potential to unlock such applications for brain cancer.

### Guiding plasma analysis using tumour-tissue DNA mutations

A practical approach to improve tumour-signal detection in cfDNA is to track multiple mutations simultaneously. Standardised panels are conceptually limited by the fraction of the genome covered, the depth of sequencing and the number of starting cfDNA molecules in the sample. By using tumour-informed (or personalised) sequencing, mutations and alterations initially identified in the tumour can be sought. This approach boosts coverage across defined loci and thus increases the chances of low tumour-fraction variants being detected at modest cost. A larger number of variants, compared with standardised panels, can also be followed, therefore increasing the sensitivity of sequencing without compromising specificity. Such an approach has been validated for multiple cancer types and clinical scenarios using PCR systems [42], targeted sequencing [43], capture sequencing or genome-wide sequencing [44–46]. Using tumour-guided sequencing and an algorithm called INVAR, a recent study reports that mutations were detected in 7/8 CSF, 10/12 plasma and 10/16 urine glioma samples [47]. Although tumour-guided sequencing is not suitable for diagnostic and screening purposes [44, 48], its potential to detect relapse and pseudoprogression in glioma remains unexplored. Tumour-guided sequencing could be further combined with mutational enrichment methods (via size selection or thermodynamic-based strategies), which even if they have not been tested in the glioma context, could have a strong potential for improving the monitoring of relapse in these patients [49, 50].

## Leveraging the epigenome for the analysis of glioma-derived cfDNA

Mutation analysis utilises few, highly specific, mutated regions within the genome of cancer patients for signal detection and analysis. This strategy relies heavily upon the presence of loci that are present in few DNA fragments and so, inevitably, suffers from a reduction in sensitivity, especially for cancers where relatively few copies of DNA exist within a liquid-biopsy sample (e.g. brain tumours). Sequencing the cfDNA epigenome, for example, methylated (mC) DNA, is advantageous because signatures exist across multiple, less specific, regions of difference [51, 52]. By deriving a positive signal from several characteristic differentially methylated regions, the specificity required to identify a sample as having features suggestive of a positive result is putatively generated. Importantly, the presence of multiple different DNA fragments (with no specific fragment being essential) improves sensitivity. This is also of particular relevance when dealing with scenarios whereby the number of DNA molecules is limiting (e.g. brain tumours or screening/early detection/minimal residual disease detection). Recently interest has also been directed towards alternate stable epigenomic marks such as hydroxymethylation (hmC) [53, 54] and histone modifications [55] as potential circulating epigenomic biomarkers. Moreover, the combination of multiple modified DNA and histone sites across the genome may prove additive for further boosting-test sensitivity and specificity. Currently, however, separate techniques are required for these analyses, and so, DNA quantity and total number of molecules of interest become limiting.

Rather than purely detecting the presence of cancer DNA, epigenomic marks associated with the cell of origin could be also leveraged to improve detection and classification of pathologies [32, 56]. A recent study examining CpG sites identified up to 2% of brain-derived cfDNA in the circulation of healthy individuals [57]. CpG-site analysis can also be applied to other bio-fluids (e.g. CSF) [58]. Successful strategies might therefore need to exploit cancer- and organ-specific epigenomic changes, perhaps even with identification of epigenomic marks related to treatment response and treatment failure adding benefit.

### cfDNA epigenome analysis using bisulfite conversion

Epigenomic modification of DNA can be detected through either direct or indirect means, sometimes requiring only small quantities of DNA [51]. The most common indirect technique utilises bisulfite ions to deaminate unmethylated cytosines to uracil, which are then read as thymine in the resultant amplified DNA. PCR, array and sequencing approaches can be utilised for subsequent processing. If sequencing the converted samples, computation of uracil/cytosine signal at each loci of interest provides a continuous variable of mC at that base. Bisulfite conversion provides a cheap, fast and reproducible method for mC analysis in glioma cfDNA. Lavon et al demonstrated excellent specificity (100%) although modest sensitivity (59% astrocytoma, 47% oligodendroglioma) when using methylation at the MGMT promoter as a serum marker for primary brain tumours [59]. Following bisulfite conversion of cfDNA, mC- specific PCR was performed at the MGMT locus using different primers for methylated/unmethylated fragments [59]. Estival et al. used a similar technique in matched tumour and blood samples with variable results, depending upon the assay used and the presence or absence of MGMT methylation in the primary sample [60]. Gong et al. showed benefit by analysing Alu hypomethylation (AUC 0.904) in conjunction with MGMT hypermethylation (AUC 0.962) across a cohort of 124 glioma patients [61]. Recently, Sabedot et al. used bisufite conversion of plasma-derived cfDNA followed by Epic array processing across a cohort of 149 glioma patients to discriminate with machine-learning classifier between tumour and normal samples with a sensitivity of 100% and specificity of 97.78% [62]. The same study suggested a potential utility of this methylation-based approach for tracking clinicopathological changes (e.g. progression, pseudoprogression or response to standard or experimental treatment). A recent novel approach used MR-guided focused ultrasound to ‘open’ the blood–brain barrier [63]. Meng et al. noted an increase in the amount of cfDNA and used bisulfite conversion to identify neural-lineage cells via gene-set enrichment of hypomethylated probes. In 1/9 cases (with IDH mutation), they identified the mutation through targeted dPCR [63].

However, bisulfite conversion presents limitations for cfDNA analysis. First, it necessitates harsh conditions of pH and temperature that can degrade the DNA, which when dealing with low-input quantities of cfDNA, can be limiting [64]. Moreover, the conversion can be incomplete, or there can be over-conversion, resulting in subsequent interpretation difficulties. Finally, the inability to distinguish mC from hmC, both being resistant to bisulfite-induced deamination, can further confound analysis. Despite these limitations, protocols exist that mandate only small amounts of DNA and seem to provide reliable data for use in liquid biopsy. Whole-genome bisulfite sequencing (WGBS) requires high-depth sequencing and whole-genome coverage, thus rendering it expensive. This has been partially circumvented by applying the adapter tags post bisulfite conversion (PBAT) with a resultant reduction in sequencing burden [65]. PBAT generates a PCR-free approach that can determine methylation status from as little as 125 pg of DNA and provides utility for single-cell bisulfite sequencing [66].

In order to circumvent the large quantities of DNA lost through bisulfite conversion, techniques have been developed or adopted that reduce the need for large quantities of input DNA (e.g. as required for WGBS). Reduced-representation bisulfite sequencing (RRBS) enriches for CpG islands that are putatively most representative of cell methylation status. This approach reduces sequencing costs but at the cost of omitting non-CpG methylation (e.g. intergenic and enhancer regions) [67]. It also requires high-quality DNA due to the restriction-enzyme sequence utilised to identify target regions, thus potentially limiting its efficacy in fragmented cfDNA. Whilst bisulfite conversion enables distinction between mC and C, it is unable to discriminate between mC and hmC. Given our improved understanding of the often-opposing action of hmC (when compared with mC) across the genome, it has become important to discriminate between the two modifications. Alternate techniques are involving additional base-conversion steps alongside bisulfite-conversion OxBS seq [68], TAB-seq [69] or the use of enzymatic methods of deamination [70, 71]. Moreover, recent appropriation of pyridine borane- reductive decarboxylation and deamination chemistry has provided a direct means of reading mC and hmC [72].

### cfDNA epigenome analysis using bisulfite-free methods

Bisulfite-free methods are also used and these can loosely be divided between affinity-enrichment and restriction-enzyme-mediated technologies. Affinity-enrichment methods have been developed for both methylation (MeDIP) [73, 74] and hydroxymethylation analysis [75]. MeDIP involves the use of antibody- based mC-sensitive pulldown of fragmented genomic DNA for the analysis of mC by fragmented region via PCR or sequencing [76]. Recently, this was adapted for use with cfDNA present in brain tumours and AUC was >0.71 when defining a broad range of brain tumours [77]. These plasma cfDNA methylomes combined with machine-learning analysis can discriminate common intracranial tumours with similar cells of origin, for example, by classifying IDH-mutant glioma (mean AUC = 0.82), or low-grade glioma (mean AUC = 0.93) from a group of 161 cases [77]. A similar method for hmC uses click chemistry to biotinylate glucosylated hmC residues with similar PCR or sequencing outputs [75]. These techniques are well adapted for cfDNA applications as they require low-starting input of cfDNA molecules (~5 ng). However, these techniques only determine that a modified base exists at some point along the fragment. Methylation-restriction enzymes are able to cleave unmethylated but not methylated regions. Subsequent PCR or sequencing can be performed; however, the fragmented nature of cfDNA with limited CpG-containing recognition sites can hamper methylome coverage [78].

Direct analysis of DNA without the need for conversion or amplification is a ‘holy grail’ of methylation sequencing. Long-read sequencing may also enable the phasing of data providing contextual information that may further boost sensitivity whilst reducing epigenomic noise. Recent improvements in nanopore-sequencing analysis pipelines provide an interesting counterpoint to the short-read narrative and may deliver value for analysis where DNA molecules are limited, such as those in cfDNA for brain tumours [79]. In addition, nanopore sequencing could enable the integration of genomic with epigenomic data in a fast turn-around time, which could be well adapted to liquid-biopsy application pending an increase in sensitivity [80].

### Epigenetics beyond methylation: integrating fragmentomics for liquid-biopsy applications

cfDNA is fragmented in plasma around 167 bp and multiple thereof, a size corresponding to the wrapping of DNA helix around the nucleosome. Plasma cfDNA from a variety of cancer cells tends to be shorter than the bulk of cfDNA derived from hematopoietic cells by 20–30 bp [81–83]. The sources and mechanisms of release of cfDNA in the bloodstream are multiple, including apoptosis, necrosis, senescence and other forms of cell death and active secretion [84, 85]. Beyond the size of the cfDNA fragments, more information can also be retrieved from where they are located in the genome [52], and how these fragments end [86]. For example, cfDNA fragments tend to be depleted in the promoter/TSS regions of highly expressed genes because the nucleosomes protecting them from degradation have to be moved away to enable transcription [87, 88]. As the size and position of cfDNA are not random but biologically regulated and/or altered in cancer, this is opening the development of applications focused on analysing such patterns or fragmentomics.

The methods focusing on fragmentomics, based on genome-wide paired-end sequencing, could improve existing sequencing methods, as well as mutation analysis. First, by leveraging the 20–30-bp size differences between tumour and non-tumour DNA, it is possible to either enrich for cancer signal, or filter out the false-positive signal (e.g. mutant DNA from clonal haematopoiesis). Indeed, clonal haematopoiesis-derived mutations should on average appear on longer fragments than mutations from tumour-derived cfDNA. Incorporating such information could reinforce the confidence in mutation calling when tumour fraction in plasma is extremely low (e.g. in gliomas). The potential of such a strategy has been demonstrated in a small cohort of glioma samples using INVAR [44, 48], as well as in other cancer types [89]. It is also possible to use fragment-size information to directly enrich for true positives. In vitro and in silico size selection of specific size ranges has highlighted potential enrichment in multiple cancer types and with different sequencing technologies [83, 90]. However, although size selection increases the sensitivity of detection, there is a reciprocal loss of cfDNA for analysis. Moreover, the relative median genome-wide enrichment is ~2-fold, and therefore direct application to glioma, with their low tumour fraction, might be challenging. In addition to plasma, the cfDNA fragmentation is also altered in the CSF from patients with high tumour fraction, revealing a potential for other bio-fluids [36].

Leveraging other biological properties of cfDNA-fragmentation profiles could enhance the detection of tumour-derived cfDNA. By combining copy number alterations with specific fragment-size patterns using random forest classifier, cancer can be classified with a high accuracy (AUC = 0.91 for glioma cases) [83]. Recently, Mouliere et al. demonstrated that the cfDNA-fragmentation profile from urine samples could also be leveraged using machine learning to improve the classification of gliomas from controls and other brain pathologies [47]. Beyond specific fragment-size ranges, more patterns could be inferred from cfDNA fragmentation. Various approaches using regional information [91], nucleosomal densities [87], chromatin compaction [92] and the composition in bases of fragment ends [93] have revealed potential to extract more tumour signal from genome-wide sequencing in other cancer types. Despite prior studies highlighting the prevalence of short cfDNA in plasma, longer circulating DNA also exists, some of which may represent extrachromosomal (circular) DNA (ecDNA) [94]. Despite the relative importance of ecDNA in glioma oncogenesis [95], ecDNA remains underexplored in liquid biopsy and may benefit from the use of long-read technologies.

## Discussion

The cfDNA field has benefited from a fast-growing portfolio of technologies and from a dynamic entrepreneurial ecosystem with a desire to improve non-invasive cancer analysis. Each cfDNA technology could be well adapted to one of the clinical applications of interest for CNS cancers: screening, diagnosis/classification, treatment guidance, monitoring progression and detecting relapse earlier (Table 2). However, technical advances do not always translate into the clinic for applications related to gliomas and other CNS pathologies. Despite clear progress to improve the sensitivity and specificity of cfDNA sequencing, significant improvements are required at a conceptual, technical and analytical level before clinical implementation of liquid biopsy in the CNS can be realised. In recent years, new technologies and concepts, such as epigenetic or fragmentomic characterisation alongside tumour-guided sequencing, have strong clinical potential [52]. In addition, methods leveraging the epigenome, due to their non-cancer-specific nature, have the potential to be applied to CNS pathologies beyond cancer [57]. Evidence of neuron-derived cfDNA and cerebellum cfDNA within acute neurotrauma and chronic neurodegeneration has also been generated [96]. The potential of disruptive cfDNA technologies and concepts for analysing neurodegenerative disease beyond cancer has barely started to be explored.

**Table 2:** Decisional matrix technologies vs applications for plasma analysis. ‘X’ indicates a lack of clinical utility; ‘+’ indicates the level of potential for the specific application; ‘?’ indicates unknown potential.

	Screening	Diagnosis/classification	Prognostication	Treatment selection	Monitoring (to define absence of disease)	Relapse detection (to define presence of disease)
Clinical requirements	Cheap	High specificity		Genomic information	High sensitivity	High sensitivity+
	Easily implementable	Complement pathology			High specificity+	High specificity
	Low false positive	Determine cell type of origin				
	High sensitivity					
PCR	?	++ (IDH status)	+	+	+	+
		Bettegowda et al. [13]	Fontanilles et al. [18]		Fontanilles et al. [18]	
		Muralidharan et al. [27]	Bagley et al. [16]		Muralidharan et al. [27]	
gene panel	?	++	+	++	+	+
		Schwaederle et al. [21]				
		Zill et al. [20]				
		Piccioni et al. [22]				
		Pan et al. [37]				
WES-WGS	?	+	?	+++	+	+
Tumour-guided	X	X	X	X	+++	+++
		Wan et al. [44]			Mouliere et al. [47]	
		Mouliere et al. [31]				
		Mouliere et al. [47]				
Methylome	?	++	?	++ (MGMT)	++	++
		Nassiri et al. [77]			Sabedot et al. [62]	
		Sabedot et al. [62]				
Fragmentome	?	++	?	?	?	?
		Mouliere et al., [83]				
		Mouliere et al. [47]				
TEP	?	++	?	X	++	++
		Best et al. [99]			Sol et al. [100]	Sol et al. [100]
		Sol et al. [100]				

Beyond cfDNA, multiple other tumour components (or components influenced by cancer cells) circulate within a cancer patient. Exosomes [97, 98], mitochondria [31], tumour-educated platelets [99, 100] and circulating tumour cells [7] have all been identified in the bio-fluids from patients with brain tumours. Combining with machine learning, these multiple analytes and signals could unlock some of the most challenging applications for liquid biopsy in the CNS context [9, 52]. Integrating multiple analytes with machine-learning classifier has led to an increase in detection and classification performance in other cancer types, but the potential of such approach remains underexplored in brain cancer [101, 102]. Bayesian integration of multi-omics and imaging data exhibits a potential to improve the predictive performance on longitudinal data, as demonstrated in other cancer types [103, 104]. The impact of new cfDNA technologies for the management of brain cancer using liquid biopsy can be boosted by the rise of disruptive computational and machine-learning strategies.

## Conclusion

The utility of liquid biopsy in glioma remains relatively underexplored at the view of its clinical potential. At every stage of the analysis paradigm, novel approaches to cfDNA have incrementally improved sensitivity and specificity. The advent of improved biobanking approaches has further improved substrate accessibility and reliability. We, therefore, hope that the past difficulties associated with translating cfDNA analysis in brain-tumour populations will not discourage further investigation amongst the liquid-biopsy community.

## Data Availability

Not applicable.
